# Increased Phenacetin Oxidation upon the L382V Substitution in Cytochrome P450 1A2 is Associated with Altered Substrate Binding Orientation

**DOI:** 10.3390/ijms19061580

**Published:** 2018-05-25

**Authors:** Qingbiao Huang, Grazyna D. Szklarz

**Affiliations:** Department of Pharmaceutical Sciences, School of Pharmacy, West Virginia University, Morgantown, WV 26506, USA; tommyqbhuang@gmail.com

**Keywords:** cytochromes P450, CYPs, P450 mutants, CYP1A2, T_1_ NMR, phenacetin oxidation

## Abstract

Leucine382 of cytochrome P450 1A2 (CYP1A2) plays an important role in binding and *O*-dealkylation of phenacetin, with the L382V mutation increasing substrate oxidation (Huang and Szklarz, 2010, *Drug Metab. Dispos*. 38:1039–1045). This was attributed to altered substrate binding orientation, but no direct experimental evidence had been available. Therefore, in the current studies, we employed nuclear magnetic resonance (NMR) longitudinal (T_1_) relaxation measurements to investigate phenacetin binding orientations within the active site of CYP1A2 wild type (WT) and mutants. Paramagnetic relaxation time (T_1P_) for each proton of phenacetin was calculated from the T_1_ value obtained from the enzymes in ferric and ferrous-CO state in the presence of phenacetin, and used to model the orientation of phenacetin in the active site. All aromatic protons of phenacetin were nearly equidistant from the heme iron (6.34–8.03 Å). In contrast, the distance between the proton of the –OCH_2_– group, which is abstracted during phenacetin oxidation, and the heme iron, was much shorter in the L382V (5.93 Å) and L382V/N312L (5.96 Å) mutants compared to the N312L mutant (7.84 Å) and the wild type enzyme (6.55 Å), consistent with modeling results. These studies provide direct evidence for the molecular mechanism underlying increased oxidation of phenacetin upon the L382V mutation.

## 1. Introduction

Cytochromes P450 (P450s, CYPs) are heme-containing monoxygenase enzymes, which are responsible for the oxidation of a large variety of drugs, carcinogens, and other xenobiotics in species ranging from bacteria to humans. A P450 enzyme usually has the ability to metabolize a number of different substrates, and different P450s often display overlapping substrate specificities and inhibitor susceptibilities [1,2].

The CYP1A subfamily has two isoforms, 1A1 and 1A2, which display ~72% sequence identity but exhibit different substrate specificities and inhibitor susceptibilities. CYP1A2 is one of the major hepatic CYPs exclusively in human liver (~13–15%) and metabolizes about 15% of clinical drugs [3]. CYP1A2 was first characterized as phenacetin *O*-dealkylase [4], and phenacetin *O*-dealkylation has been used as the most common marker reaction for CYP1A2 activity in the in vitro studies [5].

The crystal structure of CYP1A2 (Protein Data Bank ID: 2HI4) in complex with α-naphthoflavone (ANF) has been solved by X-ray crystallography, with the structural refinement of 1.95 Å [6]. The compact active site is closed without apparent solvent or substrate access channels. The active site cavity of CYP1A2 has a medium volume of 375 Å^3^ [6], which is larger than that of CYP2A6 (260 Å^3^) [7], but much smaller than that of CYP3A4 and CYP2C8, with cavity volumes of 1385 Å^3^ and 1438 Å^3^, respectively [8,9]. The narrow substrate binding cavity of CYP1A2 is lined by residues on helices F and I. The cavity of CYP1A2 is stabilized by strong hydrogen-bonding interactions between the side chain of Thr223 on helix F and the side chain of Asp320 on helix I. Both Thr223 and Asp320 are involved in an extensive network of hydrogen-bonded water molecules and side chains, including Tyr189, Val220, Thr498, and Lys500 [6].

The crystal structure of CYP1A2 reveals that Leu382 is located near the heme iron, and thus may play a role in determining substrate specificity. This is consistent with our previous findings that the L382V mutation alters enzyme specificity with alkoxyresorufins and phenacetin [10,11,12]. Enzyme kinetics studies have shown that the L382V mutant and other multiple mutants containing the L382V mutation displayed about 2-fold or 3-fold higher catalytic activities with phenacetin than the wild type enzyme, while other single mutants, such as T124S, T223N, V227G, and N312L, showed much lower activities [12]. In addition, molecular modeling studies have shown that the substitution of Leu by a smaller Val allowed the substrate to move closer to the heme iron, thereby promoting hydrogen abstraction and increasing P450 catalysis [12]. Although this explanation provided a plausible mechanism for changes in the catalytic activities of the mutants, no direct experimental evidence was available to support this hypothesis.

Therefore, in the current study, we used nuclear magnetic resonance (NMR) methods to further investigate the above proposition. NMR longitudinal (T_1_) relaxation measurements provide an efficient experimental method to elucidate the binding orientations of substrate within the active sites of P450 [13,14]. In general, NMR-derived T_1_ relaxation time is shortened in a distance-dependent manner when paramagnetic molecules, such as iron, are present [15,16]. Therefore, the distance of ligand protons from the heme of P450 can be estimated by calculating the difference of T_1_ relaxation times before and after P450 is bubbled with carbon monoxide and the T_1_ times of substrate protons closest to the heme iron will show more significant decreases than T_1_ times of those farther away [15,16]. We had previously reported the preferred binding orientations of phenacetin in CYP1A1 and 1A2 associated with isoform-selective metabolism using this method [17]. The results have confirmed the importance of residue 382 in CYP1A2-catalyzed oxidations and showed that a single residue substitution can dramatically affect enzymatic activity.

In the present work, we compared the distances between the protons of phenacetin and heme-iron in CYP1A2 wild type (WT) and the mutants to investigate the effects of single and multiple mutations of CYP1A2 on phenacetin *O*-dealkylation. In addition, a molecular model was provided to illustrate the different binding orientations of phenacetin in the active sites of CYP1A2 WT and the mutants using the distance constraints derived from T_1_ NMR experiments.

## 2. Results

### 2.1. Purification of CYP1A2 Enzymes

The overall yields of purified enzymes were about 25–50%, similar to those reported previously [10,11,12]. The purity of CYP1A2 enzymes was verified by sodium dodecyl sulfate polyacrylamide gel electrophoresis (SDS-PAGE) and Western blots, which indicated that the proteins were at least 95% pure. The ultraviolet/visible (UV/Vis) spectra of the purified enzymes were typical of P450s purified mainly in the low spin form, while the spectra of the Fe^2+^-CO complexes exhibited the characteristic peak at 450 nm, indicative of little or no P420 formation. The holoenzyme content of the enzymes was 40–60%, similarly as observed previously in our laboratory.

### 2.2. Interactions of Phenacetin with CYP1A2 Enzymes

**Figure 1:**
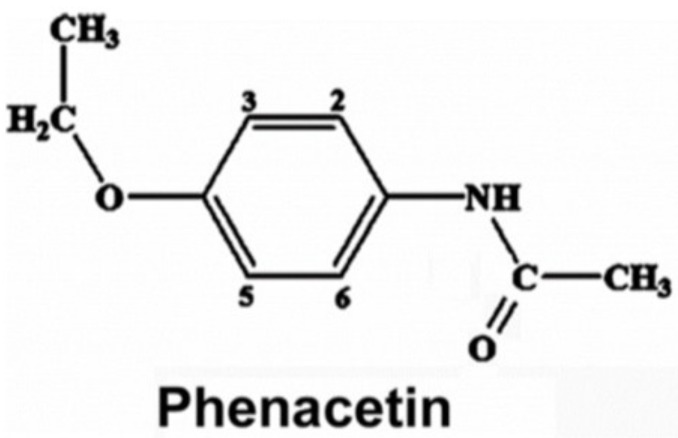
Structure of phenacetin with protons numbered as referenced in the text. Proton abstraction occurs at the –OCH_2_– group, which is the oxidation site.

**Figure 2:**
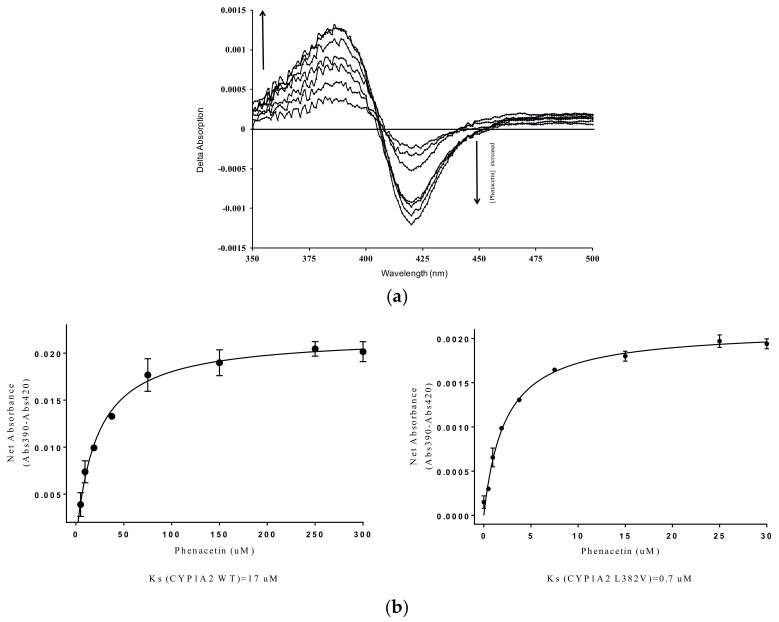
Phenacetin binding determined for CYP1A2 wild type (WT) and the mutants by ultraviolet/visible (UV/Vis) spectroscopy. (**a**) Spectral binding curves for phenacetin bound in CYP1A2 L382V. A type I spectrum is evident with a peak at ~390 nm and a trough at ~420 nm. CYP1A2 WT, N312L and L382V/N312L have similar UV/Vis binding spectra (not shown); (**b**) Binding curves for phenacetin in CYP1A2 WT and the L382V mutant and the K*_S_* values derived from fitting of the data to Equation (1).

The chemical structure along with the proton numbering scheme used for phenacetin is shown in Figure 1. The P450 absorbance spectrum is sensitive to ligand binding and solution conditions, and is composed of overlapping spectra of low spin (S = 1/2) and high spin (S = 5/2) states of the heme [18]. The addition of different concentrations of phenacetin to purified CYP1A2 WT and mutants led to an increase of their Soret peak at 390 nm and a decrease of a band at 417 nm, as shown in Figure 2A. Thus, for all enzymes, we observed a typical Type I spectrum, which indicates a change of the spin state of the heme iron from low spin (S = 1/2) to high spin (S = 5/2) due to the binding of the substrate to the protein in close proximity of heme [19]. The dependence of UV/Vis absorption changes on the concentration of the substrate was used to calculate the spectral binding constants, Ks, for the P450-phenacetin complexes at 27 °C. Table 1 presents the Ks values for phenacetin binding with CYP1A2 WT and the mutants. The Ks values for N312L, L382V, L382V/N312L mutants were lower than that of the WT enzyme (Table 1, Figure 2B).

### 2.3. Determination of Spin State in CYP1A2 Enzymes

The UV/Vis spectra (wavelength 320–500 nm) of CYP1A2 WT and the mutants can be deconvoluted into a low-spin component (~416–420 nm), a high-spin component (~390–405 nm), and δ bands (~360 nm). The concentrations of phenacetin used in the determination of spin state were identical to those used in NMR T_1_ studies. The percentages of low and high spin calculated from the Soret bands of CYP1A2 WT and the mutants in the absence and presence of substrate are shown in Table 2. In the absence of substrate, CYP1A2 WT and the mutants, L312N, L382V, and L382V/N312L, existed primarily in the low spin state (93–97% low spin). The addition of phenacetin increased the percentage of high-spin enzyme approximately 2- to 3-fold. It is critical to know these relative percentages for interpreting NMR data because the increasing percentage of high-spin state results in paramagnetic broadening and shifting [20,21].

**Figure 3:**
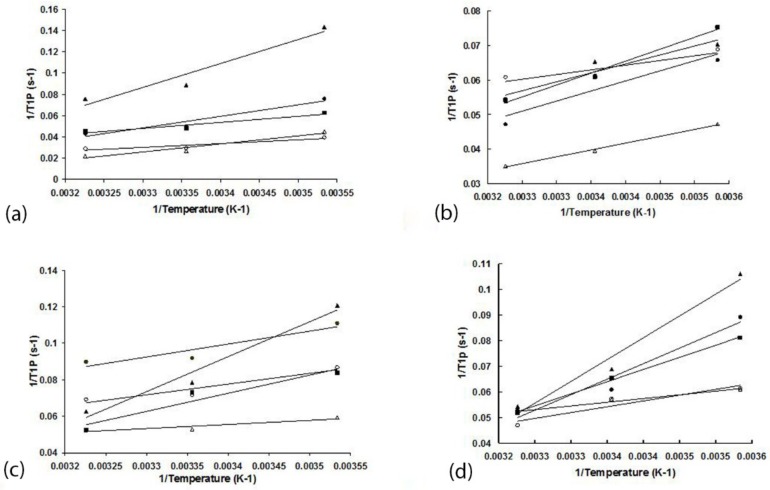
Temperature dependence of T_1P_ of phenacetin protons in the presence of CYP1A2 WT (**a**), N312L (**b**), L382V (**c**), and L382V/N312L (**d**) mutants. Positive slopes indicate that the substrate bound in the active site is in fast exchange with the surroundings. ●, phenacetin protons H2/6; ○, H3/5; ▲, CH2; △, COCH3; ■, CH3.

To verify fast-exchange conditions, the temperature dependence of the T_1_ relaxation of the substrate protons was used. The double reciprocal plots of T_1p_ versus temperature for CYP1A2 WT, L312N, L382V, and L382V/N312L mutants are shown in Figure 3. The positive slopes in the double reciprocal plot of T_1_p versus temperature indicate that the fast exchange condition is being met. Double reciprocal plots of T_1,Fe3+_ or T_1,Fe2+-CO_ versus temperature also have positive slopes (data not shown).

### 2.4. Nuclear Magnetic Resonance (NMR) T_1_ Experiments

**Figure 4:**
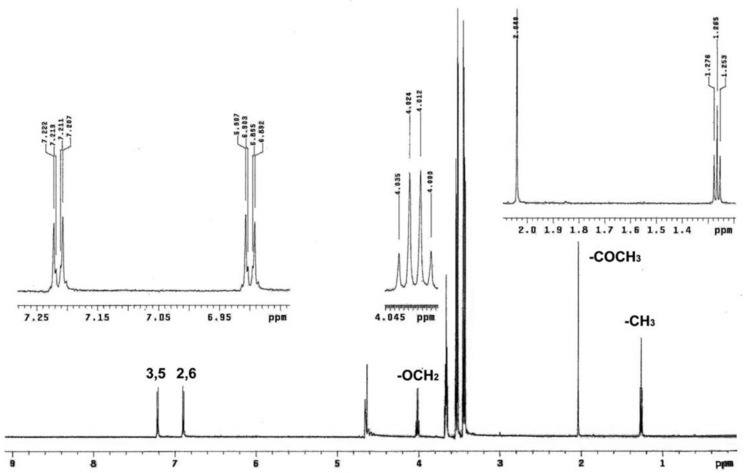
Nuclear magnetic resonance (NMR) spectrum of the protons of phenacetin obtained under conditions used for T_1_ measurements. The numbering scheme used is the same as shown in Figure 1.

The ^1^H NMR spectrum of phenacetin consists of five well-resolved signals (one NMR peak, one triplet, one quadruplet, and two doublets) and some additional peaks reflecting contamination (Figure 4). From the splitting and the relative area of the peaks, one doublet centered at 7.21 ppm was assigned to the protons at positions 3 and 5 on the phenyl ring, while the other doublet with the same peak area but centered at 6.90 ppm was assigned to the protons at positions 2 and 6 on the phenyl ring. In contrast, –COCH_3_, –CH_3_ and OCH_2_– groups of phenacetin formed a singlet (centered at 2.04 ppm), a triplet (centered at 1.26 ppm), and a quadruplet (centered at 4.02 ppm) in the NMR spectrum, respectively. The average distances between the substrate protons and the heme iron for phenacetin with each of the enzymes, CYP1A2 WT, N312L, L382V, and L382V/N312L mutants, were obtained from T_1_ relaxation experiments (Table 3). T_1_ relaxation times decreased for all protons of phenacetin in the presence of enzymes compared to those measured in the presence of enzymes and carbon monoxide. T_1_ relaxation times showed a substantial difference between the samples with and without carbon monoxide. All the aromatic protons of phenacetin are nearly equidistant from the heme iron (6.34–8.03 Å). However, the distance between the proton of the –OCH_2_– group, which is abstracted during phenacetin oxidation, and the heme iron is much shorter in the L382V mutant (5.93 Å) and L382V/N312L mutant (5.96 Å) than that in WT (6.55 Å). In contrast, the distance between the proton of the –OCH_2_– group and the heme iron is longer in the N312L mutant (7.84 Å) than that in WT, consistent with previous molecular modeling results. In addition, the distances obtained by averaging the 20 lowest energy conformations of the phenacetin are also presented in Table 3. The distances between the protons of phenacetin and heme iron obtained from molecular modeling were about 1 Å longer, but still correlate well with T_1_ data.

### 2.5. Position of Phenacetin Relative to Heme in CYP1A2 Wild Type (WT) and Mutants

**Figure 5:**
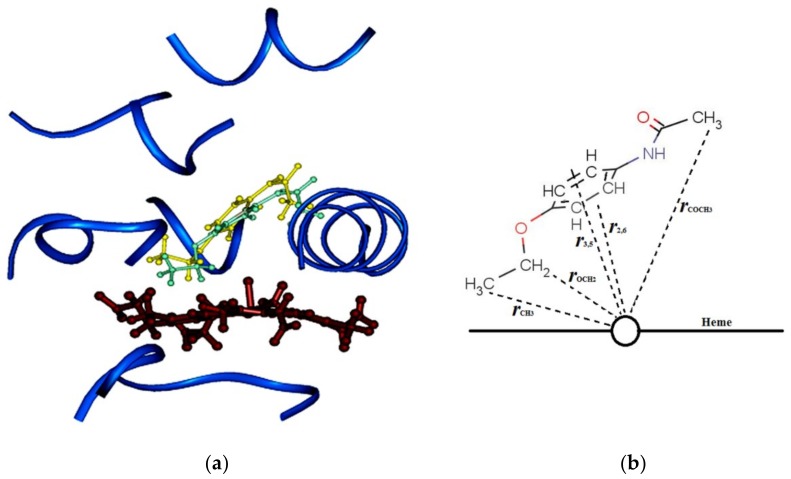
Binding of phenacetin in the active site of CYP1A2 WT and L382V. (**a**) A comparison of phenacetin binding orientations in CYP1A2 WT (substrate shown in yellow), and in CYP1A2 L382V (substrate shown in green). The enzymes were superimposed using the protein backbone as a basis; for clarity, the mutant enzyme is not displayed; (**b**) Cartoon representation of (**a**) to clearly show the distances of protons to heme iron. The heme is labeled and shown as a circle and a line, the distances of protons of phenacetin to heme iron are listed and represented by dotted lines.

3D models consisting of the substrate phenacetin and CYP1A2 WT and various mutants that correspond to the NMR-derived proton-heme distances listed in Table 3 were constructed. The enzyme-substrate interaction energies for various substrate conformations were never higher than 5 kcal/mol. Figure 5a displays the binding orientations of phenacetin within the active sites of CYP1A2 WT and the L382V mutant. Additionally, in order to clearly identify each atom in phenacetin and the distances between protons of phenacetin and heme iron, the cartoon diagram is presented as Figure 5b. As shown in the figures, phenacetin displays similar binding orientations in both CYP1A2 WT and L382V mutant. However, based on the final position after the restraint molecular dynamics (MD) run, the protons of –OCH_2_ group of phenacetin, the site of metabolism, are closer to the heme-iron in the L382V mutant (6.95 Å) than the WT (8.01 Å).

## 3. Discussion

The objective of the present study was to explore the structural basis for changes in CYP1A2 activity upon the substitution of residue Leu382 using phenacetin as a probe. Previous work has demonstrated that all CYP1A2 mutants that contained the L382V substitution (L382V, L382V/T223N, L382V/N312L, L382V/T223N/N312L, L382V/T124S/N312) displayed much higher phenacetin oxidation activities than CYP1A2 WT, with K_cat_ values 3-fold higher, in contrast to other mutants (T124S, T223N, V227G, and N312L), for which K_cat_ decreased [12]. Further stoichiometry studies suggested that the CYP1A2 L382V mutants were more efficient in coupling NADPH to the product, and displayed less uncoupling to water, leading to the increased overall coupling efficiency of the enzyme [12]. Molecular modeling indicated that L382V substitution increased the volume of the active site near the heme and allowed phenacetin to move closer to the heme, which promotes hydrogen abstraction [12].

In order to provide direct evidence to support our previous findings, we investigated the orientation of substrate phenacetin in the active site of CYP1A2 WT and its mutants using NMR T_1_ relaxation measurements. This allowed us to calculate the distance between phenacetin protons and heme iron in various CYP1A2 enzymes. Except for the –OCH_2_– group, the substrate proton-heme iron distances for CYP1A2 WT, L382V, N312L, and L382V/N312L were very similar, ranging from 6.1 to 8.2 Å. These values are similar to the distances between hydroxylation sites and the heme iron reported for other P450 enzymes [15,17,22,23,24,25,26]. The distance between the protons of the –OCH_2_– group and heme iron in CYP1A2 N312L was about 7.84 Å, which is much longer than that in CYP1A2 WT (6.55 Å). In contrast, the distances in CYP1A2 L382V and L382V/N312L were 5.93 Å and 5.96 Å, respectively, much closer to the heme iron than those of the WT enzyme (Table 3). Interestingly, the same distances obtained from molecular modeling were 1–2 Å longer than those from the NMR data. The reason, in part, might be because the distances calculated from MD are based on a very small subset of orientations, while the distances calculated from the NMR T_1_ data represent a large number of possible orientations. However, these values should be very similar if MD run time is sufficiently extended.

Previously, we had successfully used the T_1_ NMR methodology to estimate the distances between the –OCH_2_– protons of the ethoxy group (site of phenacetin *O*-deethylation) and the heme iron in both CYP1A1 and CYP1A2 WTs [17]. The calculated distances were shorter in CYP1A2 WT than in CYP1A1 WT, indicating a more efficient metabolism of phenacetin by CYP1A2. Taken together, our findings from the previous and current studies on CYP1A2 WT and the mutants provide a rationale for the efficient phenacetin *O*-dealkylation by CYP1A2 WT and 1A2 mutants containing the substitution of Leu-382 with a small Val. In contrast, CYP1A1 and CYP1A2 N312L metabolized phenacetin with a very low efficiency, as shown in our previous studies [12].

The NMR T_1_ relaxation measurements were also used to examine the orientation of other substrates within the active site of human CYP1A2. Regal and Nelson investigated the positioning of caffeine in CYP1A2 and found that the averaged distance for the N_3_ group was shorter than those for N_1_ and N_7_ groups [15], consistent with the known preference of the enzyme, which oxidizes caffeine at the N_3_ position [27,28]. However, Regal and Nelson calculated the relevant distances using an equation, $r=C{\left[T_{1P}\alpha_{m}f\left(\tau_{c}\right)\right]}^{1/6}$, which did not take into account the fact that the spin state may change upon substrate binding and, therefore, the value of C may be incorrect [29]. Thus, the authors had to provide a range of distances, representing the change in the spin state of CYP1A2 ranging from 100% low spin to 100% high spin. The modified equation, $r={\left[9.78\times 10^{16\;}T_{1P}\alpha_{m}S\left(S+1\right)\tau_{c}\right]}^{1/6}$, used in the present work, shown as Equation (2), takes into the account the constant changes of spin state, and has been widely used to calculate the distances in NMR T_1_ measurement in more recent studies [12,29,30].

A bias in the calculations leading to an overestimation of the distances can be introduced if the exchange between the free and bound ligands is not fast enough to neglect the residence time for the protons near the paramagnetic site [31]. To test the fast exchange condition, three different temperatures: 283, 298, and 310 K were adapted to perform T_1_ relaxation measurements. The slopes 1/T_1,Fe3+_, 1/T_1,Fe2+-CO_, and 1/T_1p_ versus 1/temperature are all positive and show a good linear relationship (R^2^ > 0.95) (Figure 3), which suggests that fast exchange exists.

NMR T_1_ relaxation measurements have been often used for investigating substrate orientation in various P450s. However, it has not been applied as extensively as other spectroscopic techniques due to the requirements for large amounts of highly purified enzymes and sufficiently water-soluble substrates.

The interaction between phenacetin and CYP1A2 WT and the mutants resulted in a type I binding spectrum (Figure 2a for CYP1A2 L382V, other data not shown), which indicates the interaction is a competitive binding, in contrast to ligation of the inhibitor to the heme iron (type II binding) or the formation of a metabolite inhibitory complex (MIC) by mechanism-based inhibitors (type III binding) [32]. Although local single or multiple mutation of CYP1A2 did not change the interaction type of substrate with enzyme, the binding capability altered significantly upon mutation. Ks values indicate that phenacetin binds much tighter to CYP1A2 L382V and L382V/N312L than in the WT and N312L (Table 1), as well as CYP1A1 [17]. This may indicate that phenacetin, and especially the oxidation site, moves much closer to the heme iron in CYP1A2 L382V and L382V/N312L to form a more stable complex.

Similar to previous findings with CYP1A1 WT and CYP1A2 WT [17], the ligand-free CYP1A2 WT and the mutants exhibited a high percentage of low spin state (90–95%), indicating that a water molecule forms a sixth axial ligand of the Fe^3+^ in the substrate-free form. The percent of high spin P450 was substantially increased during co-incubation with the substrate phenacetin (Table 2) due to the displacement of the water ligand by the substrate. Although the percentages of high spin for CYP1A2 WT (17%), L382V (16%), and L382V/N312L (11%) were higher than for N312L (8%), the relationship between binding affinity and the ability to increase the percentage of high spin state of the enzyme is still unclear.

In summary, the substitution of Leu382 with a small Val in CYP1A2 led to the substrate phenacetin moving closer to the heme, with the site of metabolism closer to the heme iron than in the WT enzyme. Spectral binding studies revealed that the mutation of L382V significantly increased the affinity of the mutants for phenacetin. These results demonstrate that both the distance between protons of phenacetin and the heme iron, as well as the change in substrate binding affinity are likely responsible for altering the catalytic efficiency of the enzyme, which is consistent with our previous findings regarding enzyme kinetics and stoichiometry, as well as predictions from molecular modeling studies [12].

## 4. Materials and Methods

### 4.1. Materials

Phenacetin, dilauroyl-l-3-phosphatidyl choline (DLPC), sodium dithionite, polyvinyl pyrolidone (PVP) and D_2_O were purchased from Sigma-Aldrich (St. Louis, MO, USA). Potassium phosphate and EDTA were purchased from Fisher Scientific (Pittsburgh, PA, USA). Emulgen^®^ and Chaps^®^ were obtained from the Chemical Division of the KAO Corporation (Tokyo, Japan) and EMD Biosciences (Lajolla, CA, USA), respectively. Deuterium oxide (D_2_O, 99.9%) was obtained from Cambridge Isotope Laboratory (Andover, MA, USA). Centricon 10 kDa MW cut-off filters were purchased from Millipore Corporation (Billerica, MA, USA). Carbon monoxide was obtained from Mountain State AirGas (Morgantown, WV, USA). All other chemicals were of analytical grade and were purchased from standard commercial sources.

### 4.2. Protein Expression and Purification

CYP1A2 WT, two single mutants N312L and L382V, and a multiple mutant L382V/N312L, were expressed and purified according to previously established methods [10,11,12]. Briefly, the His-tag-containing CYP1A2 WT and mutants were expressed in *Escherichia coli* DH5α cells, and the enzymes were purified by affinity chromatography with Ni-NTA agarose. During purification, 5 mM caffeine was added to all the buffers to stabilize the enzyme. Subsequently, caffeine was removed completely from the preparation at the ultrafiltration stage [12]. Rat P450 reductase was expressed and purified as described earlier [10,33]. The purified enzyme fractions were aliquoted and stored at −80 °C for further use.

### 4.3. Binding Constant Determination

The interactions between the substrate phenacetin and purified CYP1A2 WT and mutants were studied by difference visible spectroscopy [34] on a Beckman DU640 spectrophotometer (Beckman Instruments Inc., Fullerton, CA, USA). Solutions (800 µL) contained 0.5 µM CYP1A2 enzymes in 100 mM phosphate buffer (20% glycerol and 0.1 mM EDTA, pH 7.4). The spectrophotometer was set to record spectra from 350 to 500 nm. The temperature was held constant at 27 °C. Two microliters of different concentrations of solutions of phenacetin in methanol were added to the sample cuvette, and the same volume of methanol was added to the reference, and UV/Vis spectra were then recorded. The spectral binding constant (Ks) was derived from fitting of the data to Equation (1) using GraphPad Prism 7 (GraphPad Software Inc., La Jolla, CA, USA):

(1) $$\Delta A=\frac{B_{\max}\times \left[S\right]}{K_{s}+\left[S\right]}$$

where ΔA represents absorbance difference, B_ma*x*_ is the maximum absorbance difference extrapolated to infinite ligand concentration, and [S] is the substrate concentration.

### 4.4. Spin State Determination

The percentages of high and low spin in CYP1A2 enzymes upon binding of phenacetin were determined as previously described [32]. Spectral titrations with phenacetin in CYP1A2 enzymes were performed by spectral scanning between 320 and 500 nm, with substrate concentrations identical to those in NMR studies. By using multiple Gaussian curve fitting with the OriginPro v8 package (OriginLab Corporation, Northampton, MA, USA), absorbance spectra were deconvoluted into three components: a low-spin component, a high-spin component, and the broad δ-band. The three components (δ-bands, low spin, and high spin) were used to fit data for phenacetin with CYP1A2 enzymes and estimate percentages of low spin.

### 4.5. Enzyme Preparation for NMR

The procedure of sample preparation and incubation has been described in detail previously [17]. Briefly, 1% (*w*/*v*) PVP in 100 mM deuterated phosphate buffer was prepared and applied to the surface of 10 kDa MW cutoff Centricon filter. The enzyme preparation was exchanged and concentrated against 100 mM deuterated phosphate buffer over the Centricon filter by centrifugation (1000× *g*, 4 °C) for 3~4 times. The final enzyme preparation contained less than 1% glycerol and ~20 μM CYP1A2.

### 4.6. NMR Spectroscopy

NMR T_1_ relaxation studies were carried out on a Varian Inova NMR Spectrometer (Varian Inc, Palo Alto, CA, USA) operating at 600 MHz, internally locked on the deuterium signal of the solvent, deuterium oxide (D_2_O), as described previously [17,29]. Signals were referenced internally to hydrogen deuterium oxide (HDO) peak at 4.8 ppm. A standard inversion recovery sequence (d_1_-180°-d_2_-90°) was utilized, along with presaturation of the residual HDO signal. The 90° pulse width was calibrated on each sample. The preacquisition delay d_1_ was set to 10 × T_1_ (40 s) of the longest relaxation time. The NMR spectra associated with at least 10 d_2_ values were needed. Line broadening and the Gaussian fitting function were used for precise calculations of T_1_ values for the protons. The value of the longitudinal relaxation time was obtained from the Varian software by using a nonlinear least-square fitting of the peak height as a function of the delay d_2_. T_1_ values were calculated from the substrate solutions without the enzyme, and after the addition of the enzyme. The T_1 (ferrous-CO)_ of the ligand protons were measured after in situ conversion of the enzyme to its diamagnetic ferrous carbonyl complex (Fe^2+^-CO) by bubbling carbon monoxide for 15 min, followed by adding ~1 mg of sodium dithionite. The sample was allowed to equilibrate for 30 min before T_1 (ferrous-CO)_ measurement. The final T_1 (ferrous-CO)_ measurement of the ligands in presence of the Fe^2+^-CO complex and the T_1_ of the enzyme-free ligand were nearly identical, which indicates very little paramagnetic contribution due to impurities. The integrity of the enzyme was assayed by measuring the UV/Vis spectra of samples maintained under similar conditions. No significant P420 formation was observed throughout the experiment.

To validate the fast-exchange conditions, the temperature dependence of T**_1P_** was studied at three different temperatures (283, 298, and 310 K). Experiments involving enzyme titration studies with CYP1A2 WT and the mutants were performed with phenacetin as a substrate. Concentrated enzyme was added to the substrate in small increments, and the T**_1_** measurements were performed after each addition.

### 4.7. Proton-Heme Iron Distance Calculations

A more precise method for the distance (*r*) calculation using spin-state data was adopted, described in details earlier [17,29]. Briefly, the calculation for distance can be written as Equation (2):

(2) $$r={\left[9.78\times 10^{16\;}T_{1P}\alpha_{m}S\left(S+1\right)\tau_{c}\right]}^{1/6}$$

The distance is given by *r*. The tumbling coefficient *τ_c_*_,_ calculated by measuring T_1P_ at several magnetic field strengths, represents the correlation time of the dipolar interactions of the protein in solution [16]. An estimate for *τ_c_* of CYP1A2 is 3.38 × 10^−10^ s^−1^, as reported previously [15]. T_1P_ is the portion of T_1_ due to paramagnetic affects alone and is given by Equation (3):

(3) $$\frac{1}{T_{1P}}=\frac{1}{T_{1,{\mathrm{Fe}}^{3+}}}-\frac{1}{T_{1,{\mathrm{Fe}}^{2+}-\mathrm{CO}}}$$

assuming that all of the diamagnetic contribution is represented by 1/T_1,Fe2+-CO_ [15]. This assumption appears to be generally valid when used in many similar studies [25,35]. The parameter α_m_, the fractional binding coefficient, is obtained by the equation $\alpha_{m}=\frac{\left[P450\right]}{K_{S}+\left[S\right]}\;$ under conditions of fast exchange when only one substrate is present [15]. Ks values determined from visible spectroscopy were utilized for the distance calculations rather than K_D_ determined by NMR for phenacetin because of the equal or slight difference between Ks and K_D_ based on Michaelis–Menten kinetics [15]. The S(S + 1) term was simplified by Equation (4) [29].

(4) $$S\left(S+1\right)=8.75f_{HS}+0.75f_{LS}$$

where *f_HS_* and *f_LS_* refer to the fractions of the high spin and low spin iron, respectively [29].

### 4.8. Molecular Modeling: General

All molecular modeling was conducted by Insight II software (Accelrys, San Diego, CA, USA) on a Silicon Graphics Octane workstation. Phenacetin was constructed and minimized using the Insight II/Builder module. Energy minimization and MD simulations were performed by the Insight II/Discover module with the consistent valence force field (CVFF). The parameters for heme containing Fe^3+^ had been described earlier [17,36,37].

### 4.9. Substrate Docking with Distance Restraints

Phenacetin as substrate was initially placed into the active sites of both CYP1A2 WT and the mutants manually to avoid steric overlaps. Automated docking of ligands was then conducted by the Insight II/Affinity module using default parameters, as previously described [10,12,17,38], except that the ferric form of the heme was used. After 20 positions (or poses) were obtained, the most energetically favorable complex was chosen and subjected to MD simulations and minimization with NMR-based distance restraints imposed. The protein backbone was tethered to its initial coordinates by a harmonic restraint force to avoid possible protein deformation resulting from restraint forces. Substrate protons were guided to NMR-derived distances from the heme iron by applying gradually strengthening harmonic restraint ($k=2-32\;\mathrm{kcal}\cdot {\mathrm{mol}}^{-1}\cdot {Å}^{-1}$) throughout 5 ps of MD. The non-bond cutoff parameter was 15 Å, and a screened Coulomb potential with a distance-dependent dielectric was used to simulate an aqueous solvent environment. After MD simulations, the final frame of the trajectory was minimized to convergence while the distance restraints were maintained during the minimization.

## Figures and Tables

**Table 1:** Spectral binding constants for phenacetin with purified CYP1A2 WT and the mutants at 27 °C. The two cuvettes contained 0.5 μM CYP1A2 WT or the mutant in 0.1 M phosphate buffer, pH 7.4, with 20% glycerol. Difference spectra were obtained after the addition of increasing concentrations of substrate to the sample cuvette.

Enzyme	Spectrum Type ($\lambda_{max}-\lambda_{min}$)	Ks
	(nm)	(μM)
CYP1A2 WT	I (390–420)	17.1 ± 0.6
CYP1A2 N312L	I (390–420)	10.2 ± 0.4
CYP1A2 L382V	I (390–420)	0.7 ± 0.1
CYP1A2 L382V/N312L	I (390–420)	3.5 ± 0.2

**Table 2:** Percentages of low spin and high spin in CYP1A2 WT and the mutants in the absence and the presence of phenacetin at 27 °C.

Enzyme (+Substrate)	Low Spin	High Spin
	%	
CYP1A2 WT (no substrate)	90	10
+Phenacetin	73	27
CYP1A2 N312L (no substrate)	94	6
+Phenacetin	86	14
CYP1A2 L382V (no substrate)	95	5
+Phenacetin	79	21
CYP1A2 L382V/N312L (no substrate)	94	6
+Phenacetin	83	17

**Table 3:** T_1_ relaxation rate-estimated distances of phenacetin protons from the heme iron of CYP1A2 WT and the mutants. Standard errors (SEs) for measurements are shown in parentheses. Errors in the T_1_ values were those reported by the fitting routine. Errors in the reported distances (r) were determined by propagation of error from the T_1_ calculation. Generally, the error is <10%.

Proton ^a^	CYP1A2 WT ^b^				CYP1A2 L382V ^c^			
	T_1,Fe3+_	T_1,Fe2+-CO_	r ^f^	R ^g^	T_1,Fe3+_	T_1,Fe2+-CO_	r ^f^	R ^g^
			Å				Å	
2,6	2.22 (0.11)	2.78 (0.05)	6.76 (0.34)	8.25	2.28 (0.11)	3.06 (0.04)	6.34 (0.31)	7.96
3.5	2.02 (0.04)	2.50 (0.01)	6.72 (0.43)	8.32	2.39 (0.22)	3.21 (0.08)	6.39 (0.28)	7.87
–OCH_2_–	1.54 (0.15)	1.85 (0.18)	6.55 (0.87)	8.01	2.43 (0.18)	4.10 (0.07)	5.93 (0.31)	6.95
–COCH_3_	1.53 (0.07)	1.62 (0.06)	7.85 (0.81)	9.44	1.64 (0.13)	1.87 (0.11)	6.79 (0.71)	8.05
–CH_3_	1.33 (0.07)	1.53 (0.06)	6.69 (0.45)	8.64	2.03 (0.13)	2.82 (0.11)	6.13 (0.89)	7.58
**Proton ^a^**	**CYP1A2 N312L ^d^**				**CYP1A2 L382V/N312L^e^**			
	**T_1,Fe3+_**	**T_1,Fe2+-CO_**	**R ^f^ (Å)**	**R ^g^(Å)**	**T_1,Fe3+_**	**T_1,Fe2+-CO_**	**r ^f^ (Å)**	**R ^g^ (Å)**
			Å				Å	
2,6	2.32 (0.10)	2.45 (0.10)	8.03 (0.42)	9.11	2.43 (0.07)	3.00 (0.06)	6.72 (0.31)	7.28
3.5	1.94 (0.09)	2.04 (0.10)	7.94 (0.43)	9.38	2.95 (0.09)	3.70 (0.11)	6.87 (0.47)	7.25
–OCH_2_–	1.62 (0.04)	1.69 (0.04)	7.84 (0.77)	8.67	1.98 (0.12)	2.90 (0.09)	5.96 (0.35)	7.24
–COCH_3_	1.54 (0.12)	1.60 (0.11)	8.19 (1.12)	9.34	2.12 (0.14)	2.59 (0.12)	6.62 (0.15)	8.01
–CH_3_	1.87 (0.12)	1.97 (0.07)	7.83 (0.52)	8.94	2.23 (0.09)	2.61 (0.11)	6.92 (0.34)	7.47

^a^ See Figure 1 for the numbering scheme of the protons for phenacetin; ^b^ [CYP1A2 WT] = 0.017 μM, [phenacetin] = 171 μM, K_S (CYP1A2 WT_) = 17.1 μM; ^c^ [CYP1A2 L382V] = 0.007 μM, [phenacetin] = 7 μM, K_S (CYP1A2 L382V)_ = 0.7 μM; ^d^ [CYP1A2 N312L] = 0.011 μM, [phenacetin] = 102 μM, K_S (CYP1A2 N312L)_ = 10.2 μM; ^e^ [CYP1A2 L382V/N312L] = 0.004 μM, [phenacetin] = 35 μM, K_S (CYP1A2 L382V/N312L)_ = 3.5 μM; ^f^ Values were calculated by $r={\left[9.78*10^{16\;}T_{1P}\alpha_{m}S\left(S+1\right)\tau_{c}\right]}^{1/6}$; ^g^ Values were obtained by averaging the 20 lowest-energy conformations selected by the docking program (See molecular dynamics (MD) Docking with Distance Restraints).

## Abbreviations

CYP	Cytochrome P450
WT	Wild Type
EDTA	Ethlenediaminetetraacetic Acid
1H NMR	Proton Nuclear Magnetic Resonance
